# Comparative Transcriptomic Analysis of Inarching Invigorating Rootstock onto Incompatible Grafts in Citrus

**DOI:** 10.3390/ijms232314523

**Published:** 2022-11-22

**Authors:** Wen He, Rui Xie, Liang Luo, Jiufeng Chai, Hao Wang, Yan Wang, Qing Chen, Zhiwei Wu, Shaofeng Yang, Mengyao Li, Yuanxiu Lin, Yunting Zhang, Ya Luo, Yong Zhang, Haoru Tang, Frederick G. Gmitter, Xiaorong Wang

**Affiliations:** 1 College of Horticulture, Sichuan Agricultural University, Chengdu 611130, China; 2 Institute of Pomology and Olericulture, Sichuan Agricultural University, Chengdu 611130, China; 3 Citrus Research and Education Center, University of Florida, Lake Alfred, FL 33850, USA

**Keywords:** inarch grafting, *Citrus junos* Sieb ex Tanaka cv. Pujiang Xiangcheng, graft compatibility, phytohormone, transcriptomic

## Abstract

Grafting is a technique that is widely used in citrus production. Graft incompatibility often occurs in the orchard. Inarching can effectively improve the vigor of incompatible grafts, but its mechanism remains poorly understood. Our previous studies investigated the scion—rootstock interaction of citrus and highlighted the role of hormonal balance and genes in abscisic acid biosynthesis regulation. To further elucidate the mechanism of inarched grafts rejuvenation, Hm/Pt combination (*Citrus maxima* (Burm.) Merrill cv. ‘Hongmian miyou’ grafted onto *Poncirus trifoliata*) were inarched with ‘Pujiang Xiangcheng’ (a novel citrus rootstock cultivar recently selected from wild *Citrus junos* populations), and comprehensive analysis was performed to compare the inarched grafts and controls. Compared with incompatible grafts, the results revealed that inarching could recover the leaf metabolism balance, including reducing starch content, increasing chlorophyll content and restoring the cell structure. Additionally, our results corroborated that hormonal balance and hormone-related genes played a central role in inarching compatibility. Furthermore, the roles of *Hsf4*, *ERF1*, *NCED3* and *PYL* were highlighted, and a model for explaining inarched grafts recovery invigoration was proposed. This study shed light on the mechanism of inarching regulation tree vigor and offered deep insights into the scion—rootstock interaction in citrus.

## 1. Introduction

Citrus is one of the most important fruit crops in the world, in terms of production. Grafting is widely used in citrus production, while some widely utilized rootstocks may still exhibit graft incompatibility in orchards [1,2,3,4]. Leaf chlorosis, discoloration, defoliation and premature senescence were commonly observed symptoms of graft incompatibility which often occur several years after grafting [5]. Graft compatibility/incompatibility between rootstock and scion is a major concern for advanced selections to be released for agricultural production. However, despite having a rich history of practice, the interactions and communications between the scion and the rootstock are highly complex and the mechanism is still far from clear [6,7,8].

It was found that graft incompatibility occurred when ‘Hongmian Miyou’ (*Citrus maxima* (Burm.) Merrill) was grafted onto trifoliate orange (*Poncirus trifoliata*) (Hm/Pt combination) [9,10,11,12]. Those grafts could be considered a potential model of the scion–rootstock interaction for citrus research [11]. Our previous studies demonstrated that incompatible grafts had a successful union formation, and the hormonal balance and genes in abscisic acid biosynthesis regulation contributed to the foliage etiolation [10,11]. Recent studies on citrus reported a positive correlation between its vegetative growth and the levels of phytohormones. For instance, dwarf and vigorous rootstocks had higher and lower levels of ABA, respectively [13,14]. Those results helped us better understand the mechanism of scion–rootstock interaction. Proper rootstock provides one mechanism by which to improve and expand citrus cultivation. However, it is impractical for adult fruit trees to replace the unsuitable rootstock with a vigorous rootstock. Inarching refers to grafting another rootstock onto the scion stem of an already grafted plant, and can be used to immediately change the original rootstock to a tolerant cultivar [15,16]. Moreover, this graft method has been applied in plant science, e.g. exploring the characteristics of nutrient absorption and detection of long-distance signals [17,18,19,20]. Although it has been successfully employed for the study of several aspects of plant metabolism and development [18,21], its application in the mechanism of graft compatibility is still limited.

We previously found that incompatible grafts inarched with ‘Pujiang Xiangcheng’, a novel citrus rootstock cultivar recently selected from wild *Citrus junos* populations [22], was able, in practice, to restore tree vigor. Therefore, inarching ‘Pujiang Xiangcheng’ is frequently used to improve absorption and utilization for existing adult trees. In this study, Hm/Pt combination was inarched with ‘Pujiang Xiangcheng’ rootstock, and physiological, biochemical, anatomical and transcriptomic analyses were comprehensively performed. Our objectives were to (1) comprehensively understand the inarching of invigorating rootstock regulated tree vigor, (2) utilize transcriptomics analysis to identify and classify the DEGs (differentially expressed genes), and (3) propose a working model for inarched graft rejuvenation. The results provided valuable information for the molecular basis of inarched grafts and enriched our understanding of the interaction between scion and rootstock in citrus.

## 2. Results

### 2.1. Double-Root System Enhanced the Growth State of Incompatible Grafts

**Figure 1:**
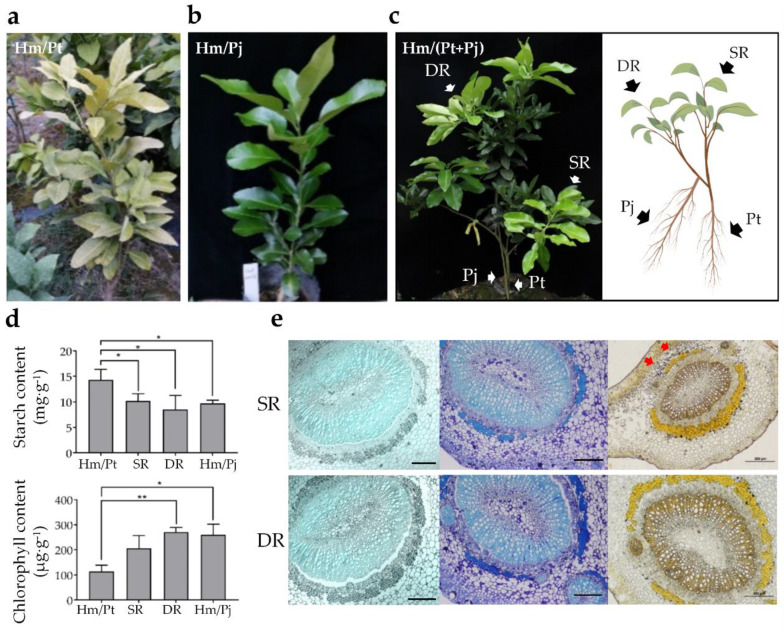
Characteristics of grafted plants. (**a**–**c**) Diagram of the grafting methods and grafted plants. (**d**) Chlorophyll content and starch content of different grafted plants. (**e**) Epifluorescence photomicrographs of phloem, starch grains dyed blue. Bars = 200 µm. Note: Hm/Pt: *Citrus maxima* (Burm.) Merrill cv. Hongmian miyou (Hm) grafted onto *Poncirus trifoliata* (Pt). Hm/Pj: ‘Hongmian miyou’ grafted onto *Citrus junos* Sieb ex Tanaka cv. Pujiang Xiangcheng (Pj). Hm/(Pt + Pj): Hm/Pt plant inarched with Pj to form single-root plant (SR) and double-root plant (DR). Asterisks represent significant differences compared to Hm/Pt (* indicate significance level of 0.05, and ** indicate significance level of 0.01). The red arrows indicate starch accumulation in (**e**).

We used *C. maxima* (Burm.) Merrill cv. Hongmian miyou (Hm) grafted onto *P. trifoliata* (Pt) as an incompatible graft combination (Hm/Pt), and Hm grafted onto *C. junos* Sieb ex Tanaka cv. Pujiang Xiangcheng (Pj) as a compatible graft combination (Hm/Pj) (Figure 1a,b) [9,10,11,12]. Etiolater trees of Hm/Pt were inarched with two-year-old Pj to form a single root system (SR) and double root system (DR) (Figure 1c). Etiolated trees could be recovered by inarching them with seedlings of Pj above their bud union lines (Figure 1a). The results showed that the vigor of the inarched plants was much stronger than Hm/Pt, and the SR and DR had similar performance (Figure 1). The chlorophyll content in leaves of DR was significantly higher than that in Hm/Pt. Furthermore, the starch content in leaves of Hm/Pt was significantly higher than that in inarched grafts (including SR and DR) and Hm/Pj (Figure 1d). We inspected the anatomical characteristics using transverse sections of the leaves. The results showed that there was no phloem plugging in all graft combinations (Figure 1e). Intriguingly, starch accumulated in the internal structure of the leaf lamina in SR, but very little starch accumulated in the internal structure of the leaf lamina in DR (Figure 1e).

### 2.2. Effect of Inarched Rootstock on Transcript Levels of Different Grafted Plants

**Figure 2:**
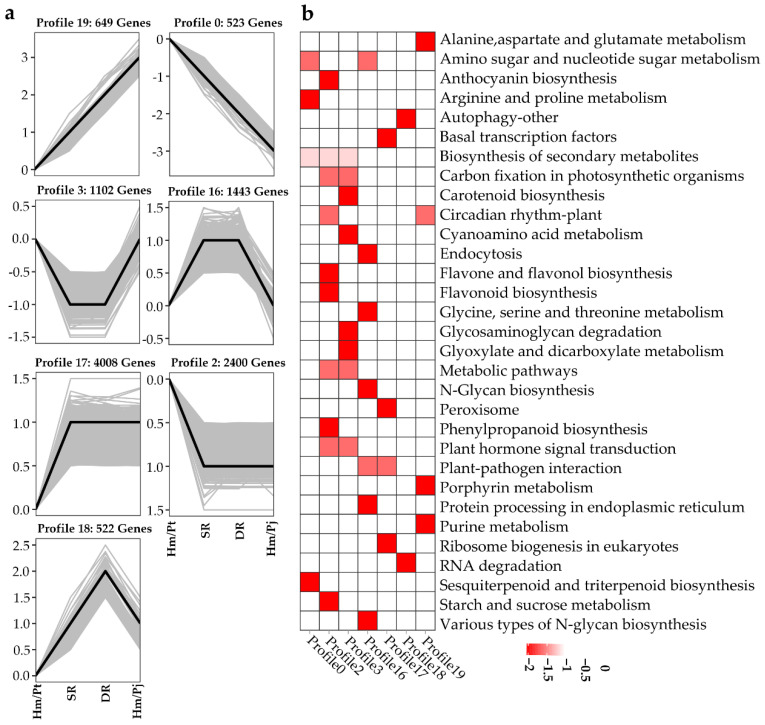
Gene expression trend analysis. (**a**) Gene expression trends. (**b**) KEGG enrichment analysis of all profile. Each line in the graph a represents a gene, the pathway enrichment map is based on the top 10 pathways with *p*-value less than 0.01.

RNA-Seq was carried out, as described in the methods section, to determine the transcript levels of leaves from Hm/Pt, Hm/Pj and inarched plants (including SR and DR). STEM (Short Time-series Expression Miner) implemented unique methods to cluster, compare, and visualize gene expression data to study dynamical biological processes [23]. We used the STEM software package to summarize our filtered transcript data by clustering it into 20 groups (Supplementary Figure S1); 7 distinct temporal expression patterns exhibited significant enrichment trends (Figure 2). For the continuously upregulated expression profiles, 649 genes were classified into profile 19 (Figure 2a, Supplementary Figure S2). The KEGG (Kyoto Encyclopedia of Genes and Genomes) pathway analysis results showed that most of the genes in this profile participated in ‘Alanine, aspartate and glutamate metabolism’, ‘Purine metabolism’, ‘Porphyrin metabolism’ and ‘Circadian rhythm—plant’ (Figure 2b). For the persistently downregulated expression profiles, 523 genes were classified into profile 0 (Figure 2a, Supplementary Figure S2). The results showed that most of the genes in this profile participated in ‘Amino sugar and nucleotide sugar metabolism’, ‘Biosynthesis of secondary metabolites’, ‘Arginine and proline metabolism’ and ‘Sesquiterpenoid and triterpenoid biosynthesis’ pathways (Figure 2b).

Phytohormones regulate every aspect of plant development and response to biotic and abiotic stresses [24]. The results showed that ‘plant hormone signal transduction’ genes were enrichment in profile 2 and profile 3 (Figure 2b). Moreover, compared with inarched grafts (including SR and DR) and Hm/Pj, the genes in Hm/Pt showed different expression patterns in profile 2. In addition, among 38 hormone-related genes in profile 2, the majority of these genes were 17 auxin-responsive protein genes, 4 auxin-induced protein genes, TIR genes and two GH3 family genes, respectively (Supplementary Table S3). All the above information revealed the pattern of candidate genes’ expression and laid some foundation for further confirmation of the graft incompatibility genes and detailed analyses of molecular mechanisms regulating graft compatibility in *Citrus*.

### 2.3. Identification of DEGs between Different Graft Combinations

**Figure 3:**
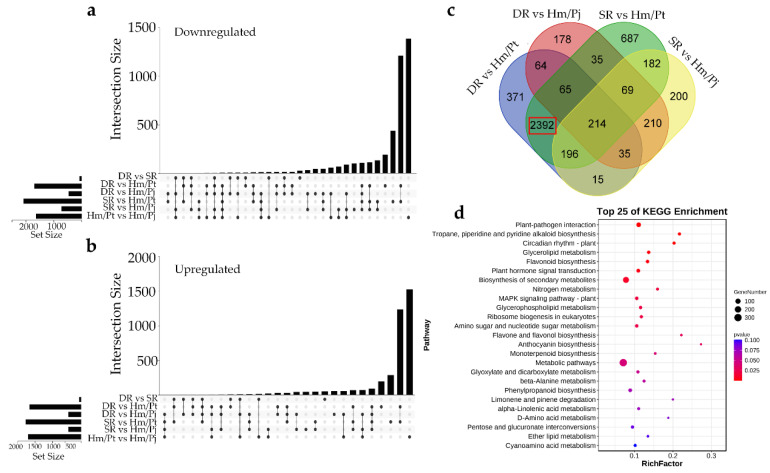
DEG analysis between different graft combinations. (**a**,**b**) Upset plots of the number of downregulated and upregulated genes (cut-off threshold, |log2(FC)|  ≥  2; *p*-value < 0.05) demonstrating different temporal expression patterns (top bar graphs). The total numbers of upregulated and downregulated genes are shown on the left. (**c**) Venn diagram of DEGs in all graft combinations. (**d**) KEGG pathways for 2392 DEGs.

To better understand the molecular basis of the metabolic differences detected in the different graft combinations, the DEGs between Hm/Pt, Hm/Pj and inarched plants (including SR and DR) were identified. The results showed that, compared with the Hm/Pt, the number of DEGs of DR were 1660 upregulated and 1692 downregulated, and the number of DEGs of SR were 1763 upregulated and 2077 downregulated, respectively (Figure 3a,b). In order to analyze the mechanism of easing the symptoms by inarching with ‘Pujiang Xiangcheng’, we used Hm/Pj as the control. There were 2392 DEGs between incompatible grafts (Hm/Pt) and inarched grafts (SR and DR) (Figure 3c). Our analysis focused on the hormones that showed relevant differences in the inarched (SR and DR) and Hm/Pt combinations; 31 DEGs were enriched in ‘Plant hormone signal transduction’ pathway, including 10 auxin-related genes, 5 ABA-related genes and 5 ethylene-related genes (Figure 3d, Supplementary Table S4).

Furthermore, there were 64 DEGs between DR with Hm/Pt and Hm/Pj, and 182 DEGs between SR with Hm/Pt and Hm/Pj, respectively (Supplementary Figure S3). This may have been the interaction between the two rootstock signals resulting in the gene expression in DR intermediately between those of Hm/Pt and Hm/Pj. Additionally, the statistical analysis results showed that the number of DEGs were relatively few between DR with Hm/Pt and Hm/Pj. KEGG enrichment analysis was also performed, and several DEGs were related to the synthesis of plant hormone signal transduction, such as *JAZ* (*Cg7g022240*), *AUX1* (*Cg3g016080*), *AUX*/*IAA* (*Cg4g006940*), *GH3* (*Cg8g003480*) and *PP2C* (*Cg8g023080*). The results showed that the interaction between scion and rootstock could change the gene expression of the whole plant.

### 2.4. Identification of DEGs between Single-Root and Double-Root Grafted Plants

**Figure 4:**
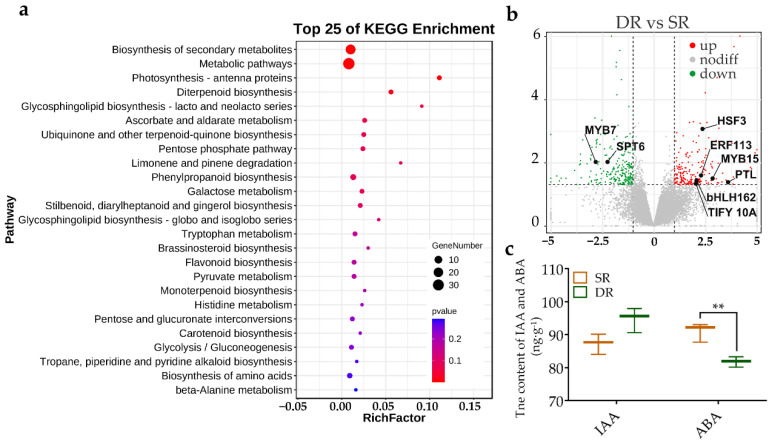
Different analysis among single-root and double-root grafted plants. (**a**) KEGG enrichment analysis of DEGs. (**b**) Volcano plots of DEGs that were upregulated (red) or downregulated (green); transport factor and hormone related genes labeled. (**c**) The content of indole-3-acetic acid (IAA) and abscisic acid (ABA) (ng·g^−1^). ** indicate significance level of 0.01.

It is worth noting that there were 166 DEGs between DR and SR, which were on the same plant (Supplementary Table S5). The result of KEGG pathway enrichment showed that most of the genes in this profile participated in ‘Biosynthesis of secondary metabolites’, ‘Metabolic pathways’, ‘Photosynthesis—antenna proteins’ and ‘Diterpenoid biosynthesis’ (*p*-value < 0.05) (Figure 4a). Among those DEGs, eight genes were annotated as transcription factors (TFs) belonging to five TF families (Figure 4b). Out of 8 TF genes, 6 DEGs were upregulated (*HSF 3*, *Cg9g020350*; *ERF 113*, *Cg7g011250*; *MYB 15*, *Cg2g015060*; *PTL*, *Cg2g035410*; *bHLH162*, Cg5g004810, and *TIFY 10A*, *Cg1g010830*) and two DEGs were downregulated (*MYB* 7, *Cg6g007010,* and *SPT6*, *Cg4g015550*) (Figure 4b). In this study, an upregulated TF (*TIFY 10A*) may have played an important role in hormone signal pathways. It was also the key gene in our previous study [11]. In addition, the level of auxin was higher in DR than in SR, though there was no significant difference. The content of ABA was significantly lower in DR than that in SR (Figure 4c). Moreover, we found that two DEGs (*Cg5g042950*, *Cg1g009760*) were enriched in ‘Starch and sucrose metabolism’, which may have been responsible for starch accumulation in SR (Supplementary Table S5).

### 2.5. Identification of WGCNA Modules Associated with Hormone Pathway

**Figure 5:**
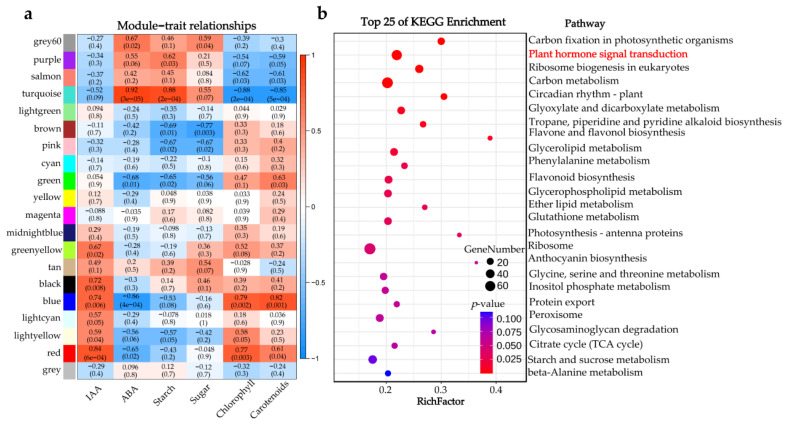
WGCNA of DEGs identified from different graft combinations. (**a**) Module-trait correlations and corresponding *p*-values. (**b**) KEGG enrichment analysis the genes in module turquoise.

A total of 19,075 expression genes were screened and selected for WGCNA analysis, resulting in 20 modules which have been marked with different colors in Figure 5. In addition, the correlation between these modules with six metabolites (IAA, ABA, starch, sugar, chlorophyll, and carotenoids) were analyzed (Supplementary Table S6). The results showed that the module ‘turquoise’ was significantly positively correlated with ABA (*r* = 0.92) and starch (*r* = 0.88), and negatively correlated with chlorophyll (*r* = −0.88) and carotenoids (*r* = −0.85). This result suggested that the 4431 genes in the module ‘turquoise’ played important roles in the correction of rootstock-induced chlorosis in citrus (Supplementary Table S7).

KEGG enrichment analysis was also performed for genes in module ‘turquoise’. The results showed that most of the genes in this profile participated in the ‘Carbon fixation in photosynthetic organisms’, ‘Plant hormone signal transduction’, ‘Ribosome biogenesis in eukaryotes’, ‘Carbon metabolism’ and ‘Circadian rhythm—plant’ pathways (top five pathways of KEGG enrichment). There were 19 DEGs (between inarched plants and Hm/Pt, Figure 3c) in ‘Plant hormone signal transduction’ pathway, including nine auxin-related genes and four ABA-related genes.

### 2.6. Validation of Candidate Genes by qRT-PCR Analysis

**Figure 6:**
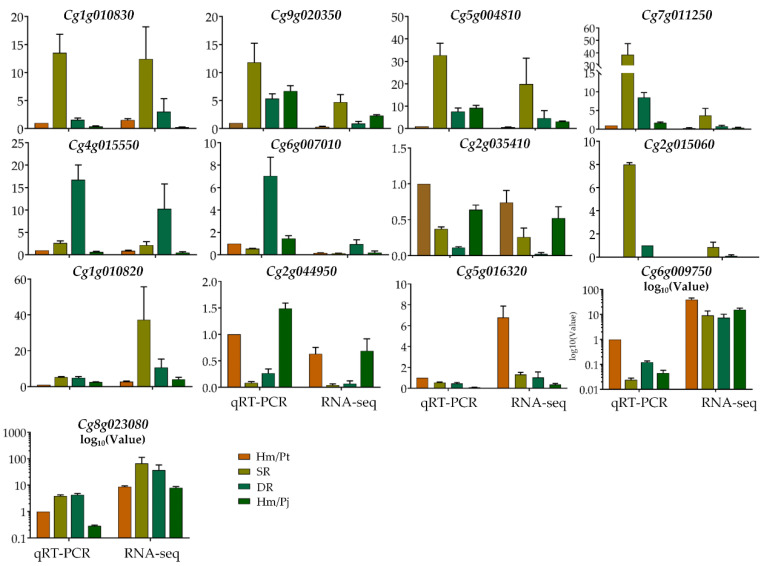
Verification of eight candidate genes’ expression in different grafts. The error bars with standard deviations are calculated from three biological replicates.

We checked eight TFs which were differentially expressed between SR and DR for expression verification by using qRT-PCR. The results showed that the qRT-PCR expression levels were generally consistent with RNA-Seq data, with a good positive correlation (*R*^2^ = 0.9363) (Supplementary Figure S4), which confirmed the reliability of the transcriptome data in the present study. Among those genes, *MYB 15* (*Cg2g015060*) was only expressed in SR and DR, not in Hm/Pt and Hm/Pj (Figure 6).

In addition, we checked the expression of six DEGs that play important roles in graft incompatibility, including *NCEDs* (*Cg5g016320*, *Cg2g044950*), *PYL* (*Cg6g009750*), *PP2C* (*Cg8g023080*) and *TIFY-10A* (*Cg1g010820*, *Cg1g010830*) (He et al., 2022). The results showed that ABA-related genes (*NCED3* and *PYL4*) were significantly decreased in inarched plants (SR and DR) and Hm/Pj, compared with Hm/Pt. Meanwhile, the expression of *PP2C* showed no difference between inarched plants (SR and DR) and Hm/Pt, but significantly decreased levels in Hm/Pj (Figure 6). Interestingly, the expression of *TIFY-10A* had a higher expression in SR than in Hm/Pt and Hm/Pj (Figure 6).

## 3. Discussion

### 3.1. Screening and Identification Candidate Graft Incompatibility Genes

Grafting has become highly important for citrus production. However, the mechanism of graft compatibility/incompatibility requires further investigation [7]. Our previous study found a potential graft model for citrus research and demonstrated the hormonal balance and genes in abscisic acid biosynthesis regulation and its contribution to foliage etiolation [11]. ‘Pujiang Xiangcheng’ is a new rootstock cultivar with resistance to multiple abiotic/biotic stresses [22] that is widely used in inarching with weak trees to improve production efficiency in an old citrus orchard. Herein, we used incompatible grafts inarched with ‘Pujiang Xiangcheng’ (which can lead to vigorous tree growth), and performed a comparative transcriptome analysis. In this study, the expression of *Hsf4* and *ERF1* showed upregulation in inarched grafts, compared with Hm/Pt (incompatible grafts) (Supplementary Figure S5). The apple (*Malus* × *domestica*) columnar gene candidate *MdCoL* and the AP2/ERF factor *MdDREB2* were able to regulate ABA biosynthesis by activating the expression of *MdNCED6/9* [25]. The *Hsf* (*Heat stress factor*) was able to connect ABA signaling and ABA-mediated stress responses [16,26]. In addition, *ERFs* also worked synergistically with the change in auxin accumulation [27]. These genes could cause the IAA content increase and ABA content decrease in inarched plants. Moreover, we detected graft incompatibility-related genes in our previous study and provided evidence for the roles of *NCED3* and *PYL* in graft compatibility [11] (Figure 6). It is worth noting that *TIFY 10A* showed high expression in inarched plants, which has been corroborated in previous studies [11]. Interestingly, *MYB 15* was only expressed in inarched plants, which could enhance sensitivity to ABA in *Arabidopsis thaliana*, leading to improved drought tolerance [28], and regulate stilbene biosynthesis in grapevine (*Vitis vinifera*) [29]. Therefore, these finds emphasize the critical importance of those TFs and genes in inarch graft compatibility. Nonetheless, the detail functions and regulation network require further research.

### 3.2. Central Role of Hormonal Balance in Graft Compatibility

Significant rootstock-induced change in hormone content has been reported in many species [30,31,32]. Our previous work validated the morphological, physiological and biochemical differences between compatible and incompatible grafts during the foliage etiolation process, and noted that hormone IAA/ABA balance triggered stress response [11]. Inarching another rootstock can enhance the nutrient concentration, change shoot hydraulic conductivity, and change water supply to the scion [16,33]. Recent works also suggested that rootstocks could change hormone levels in the scion [31,34,35]. *MdWRKY9* mediated dwarfing by directly inhibiting the transcription of the *DmDWF4* and reducing BR production in apple (*M. domestica*) [36]. ABA centric phytohormone signaling and fruit quality-related genes can be induced to different expression by different rootstocks in watermelon (*Citrullus lanatus*) [37]. In this study, there were no significant differences in the IAA content between leaves in SR and DR, but inarching by invigorating rootstock was able to significantly reduce the content of ABA (Figure 4c). There were 31 DEGs between inarched trees and Hm/Pt enriched in the ‘Plant hormone signal transduction’ pathway, including ten auxin-related genes, five ABA-related genes and five ethylene-related genes (Figure 3d, Supplementary Table S4). This indicated that IAA and ABA could play important roles in graft compatibility, consistent with our previous results [11]. The WGCNA results also supported that starch and chlorophyll content were significantly related to the expression of auxin-related and ABA-related genes (Figure 5). Overall, our study corroborated the hypothethis that IAA/ABA balance plays a central role in graft compatibility.

### 3.3. Underlying Mechanisms of Graft Compatibility in Citrus

**Figure 7:**
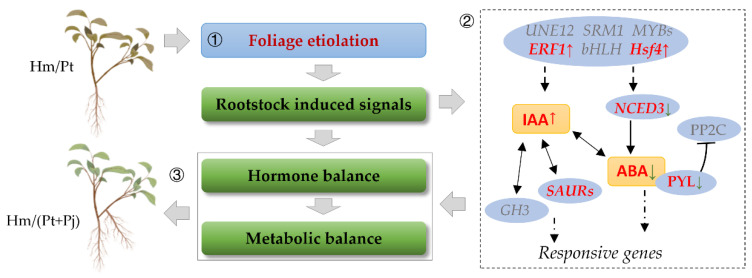
Working model of inarched grafts’ rejuvenation in citrus. ① The incompatible graft in Hm/Pt demonstrates the symptoms of the foliage etiolation. ② Inarch grafting with Pj rootstock, inducing *Hsf4* and *ERF1* upregulates expression. Those TFs cause a downregulated expression of *NCED3* and *PYL*, leading to a decrease in ABA level. Auxin-related gene expression leads to IAA level increase. Hormone levels and hormone-related genes’ expression change triggers activation of response genes expression. As the results of hormone recovery balance, metabolism gradually recovers balance, transporting the starch in leaves, increasing chlorophyll and recovering the cell structure. ③ Inarch grafting leads to vigorous growth. Note: Genes marked with red represent different expressions in this study. Genes marked with grey represent no significant difference in this study, but the so-marked genes play important roles in the foliage etiolation process in Hm/Pt [11]. The red arrow indicates upregulation and the green arrow indicates downregulation.

We refined the working model we previously proposed by using the evidence discussed above to gain insights into the citrus rootstock–scion interaction. Etiolater plant of Hm/Pt, inarched with ‘Pujiang Xiangcheng’ induced the different expression of TFs, including *Hsf4* and *ERF1*. At the same time, several genes related to the auxin-transport and auxin-responsive pathways were differentially expressed. Additionally, the IAA level was increased and ABA level was decreased. Auxin signals, including *AUX/IAA* (*Auxin/Indole-3-Acetic Acid*), *SAUR* (*Small Auxin Upregulated RNA*) and *TIR* (*Transport Inhibitor Response*), and ABA signals, including *ABF*, were regulated to coordinate plant growth and development [38,39]. This evidence, combined with our previous results, corroborated that Hm/Pt, inarched with ‘Pujiang Xiangcheng’, could recover hormone balance and metabolism balance, resulting in vigorous growth (Figure 7).

## 4. Materials and Methods

### 4.1. Plant Materials and Treatment

All plant materials were planted in the orchard of Sichuan Agricultural University, Chengdu, China. The climate was a subtropical, humid monsoon climate with 1012.4 mm annual mean rainfall and an annual mean temperature of 15.9 °C. The soil type was purple soil with pH ranging from 6.8 to 7.4. We used *C. maxima* (Burm.) Merrill cv. Hongmian miyou grafted onto *P. trifoliata* as an incompatible graft combination (Hm/Pt), and ‘Hongmian miyou’ grafted onto *C. junos* Sieb ex Tanaka cv. Pujiang Xiangcheng as compatible graft combinations (Hm/Pj) [9,10,11,12]. The two-year-old ‘Pujiang Xiangcheng’ were cut and inserted in the branch above the graft union of Hm/Pt. We named the branch with two rootstocks (Pt and Pj) as a double-root system (DR) and the branch with a single root (Pt) as a single-root system (SR) (Figure 1). After seven months of inarching or grafting, leaves of each graft combination were collected.

### 4.2. Measurement of Carotenoids, Chlorophyll, Soluble Sugar and Starch

Carotenoid and chlorophyll extraction was performed according to the method of Wellburn and Lichtenthaler [40]. Five fully mature scion leaves were taken from each combination. 0.5 g of leaf disks were cut up and suspended in 10 mL of 80% acetone and kept overnight in darkness. The absorbance of the extract was determined at 470, 663 and 645 nm with a UV2550 spectrophotometer (Shimadzu, Kyoto, Japan). Chlorophyll a (Chl a) = 12.7A_663_ − 2.69A_645_, Chlorophyll b (Chl b) = 22.9A_645_ − 4.68A_663_, total Chlorophyll (T-Chl) = Chl a + Chl b, Carotenoid (Car) = (1000A_470_ − 2.05 Chl a − 114.8 Chl b)/245. The contents of soluble sugar and starch were measured using a Plant Soluble Sugar Content Assay Kit (BC0030, Solarbio, Beijing, China) and a Starch Content Assay Kit (BC0700, Solarbio, Beijing, China) according to the manufacturer’s protocols.

### 4.3. Measurement of Endogenous Phytohormones

Three pools of leaves collected from 10 inarched or grafted plants were used for endogenous phytohormone determination. Based on our previous results [10], we focused on the level of IAA and ABA in this study. Abundant cold methanol (80%, *v*/*v*) was used to extract endogenous phytohormones. Crude extract was condensed by vacuum evaporation and hormones were re-extracted by ethyl acetate at pH 3.0 [11]. The levels of indole-3-acetic acid (IAA) and abscisic acid (ABA) were determined using enzyme-linked immunosorbent assay (ELISA, Phytohormone Research Institute, Nanjing Agricultural University, Nanjing, China), following the manufacturer’s recommendations.

### 4.4. Anatomical Observation

Samples were fixed in 2.5% glutaraldehyde in 0.03 M phosphate buffer for 24 h, then dehydrated in an ethanol series (15, 30, 50, 70 and 95%) for 90 min each. The method of sample preparation was developed and validated by He et al. [11]. The basic structure used the paraffin sections method. Starch grains were dyed by I-KI staining and observed using the Zeiss photomicroscope II (Carl Zeiss, Jena, Germany). The detection of phloem plugging was accomplished using toluidine blue and epifluorescence photomicrographs were captured using an Olympus FV3000 Laser Scanning Microscope (Olympus Inc., Tokyo, Japan).

### 4.5. RNA Extraction, RNA-Seq Library Construction

After seven months of inarching or grafting, leaves of Hm/Pt, Hm/Pj, SR and DR from inarched plants were collected and stored at −80 °C. Equal, small amounts of leaves from five inarched or grafted plants were pooled together as one replicate. Three independent replicates were carried out for RNA extraction, as described previously [10]. In addition to the previous sequencing data of Hm/Pt (NCBI: PRJNA704217) [11], we newly sequenced 9 transcriptomes, including SR, DR and Hm/Pj. Clean reads were submitted to the China National GeneBank DataBase (CNGBdb) database sequence read archive, under accession number CNP0003524. Before sequence assembly, the adapter sequences and low-quality reads were removed from the raw data (Supplementary Table S1). TopHat2 (v.2.1.1) [41] was used to map clean reads to the reference genome of *C. maxima* [42]. The number of fragments per kilobase of transcript per million mapped reads (FPKM) was calculated with the RSEM tool (v.1.3.3) [43]. The average FPKM values of the three replicates were calculated as the expression level of genes in each sample. The sets of DEGs were identified using the eBays function in the limma package (v. 3.28.14) [44] with |log2 (foldchange)| ≥ 2.0.

### 4.6. Real-Time PCR Analysis

To verify the authenticity of the transcriptional data, 13 DEGs were selected for qPCR validation. The primer sequences were designed using Primer Premier 5 software (Premier Biosoft International, Palo Alto, CA, USA) and listed in Supplementary Table S2. The qRT-PCR was performed in a 10 μL reaction volume using the TransStart^®^ Green qPCR SuperMix (TransGen Biotech Co., Ltd., Beijing, China) on a CFX96 Tounch^TM^ Real-Time PCR detection system (Bio-Rad, Hercules, CA, USA). The PCR program was as described previously [10]. The 2^−ΔΔCt^ method was utilized to calculate gene expression and *β-tubulin* gene was used as an internal control.

### 4.7. Statistical Analysis

The physiological and biochemical indexes were entered into Microsoft Excel 2020 for collation and calculation. Significant differences between grafted combinations were analyzed by Tukey’s method. Column bar and box and whiskers plots were generated using GraphPad Prism (v. 7.04).

## 5. Conclusions

In conclusion, our results supported the hypothesis that incompatible grafts’ vigor could be rapidly increased by inarching with ‘Pujiang Xiangcheng’ rootstock. The inarched plants recovered metabolism balance, including decreased starch content in the leaf, increased chlorophyll content, and enhanced recovery in the cell structure when compared with etiolater trees. This observation led us to demonstrate the important role of hormone signal in inarch graft compatibility. Furthermore, we identified several regulator genes that played vital roles during the recovery process. The detail regulation network warrants further research. Finally, we proposed a working model of inarched grafts’ rejuvenation based on the evidence observed. This study illustrated the mechanism of scion–rootstock interaction from a diverse yet comprehensive set of perspectives.

## Data Availability

Not applicable.

## Supplementary Materials

The following supporting information can be downloaded at: https://www.mdpi.com/article/10.3390/ijms232314523/s1.
