# Supplementary material for: Comparative Transcriptomic Analysis of Inarching Invigorating Rootstock onto Incompatible Grafts in Citrus
Source: Int J Mol Sci. 2022 Nov 22;23(23):14523. doi: 10.3390/ijms232314523 (PMC9735857; doi:10.3390/ijms232314523)
Supplement: Supplementary file 1 [file ijms-23-14523-s001.zip › Supplementary Figs.pdf]

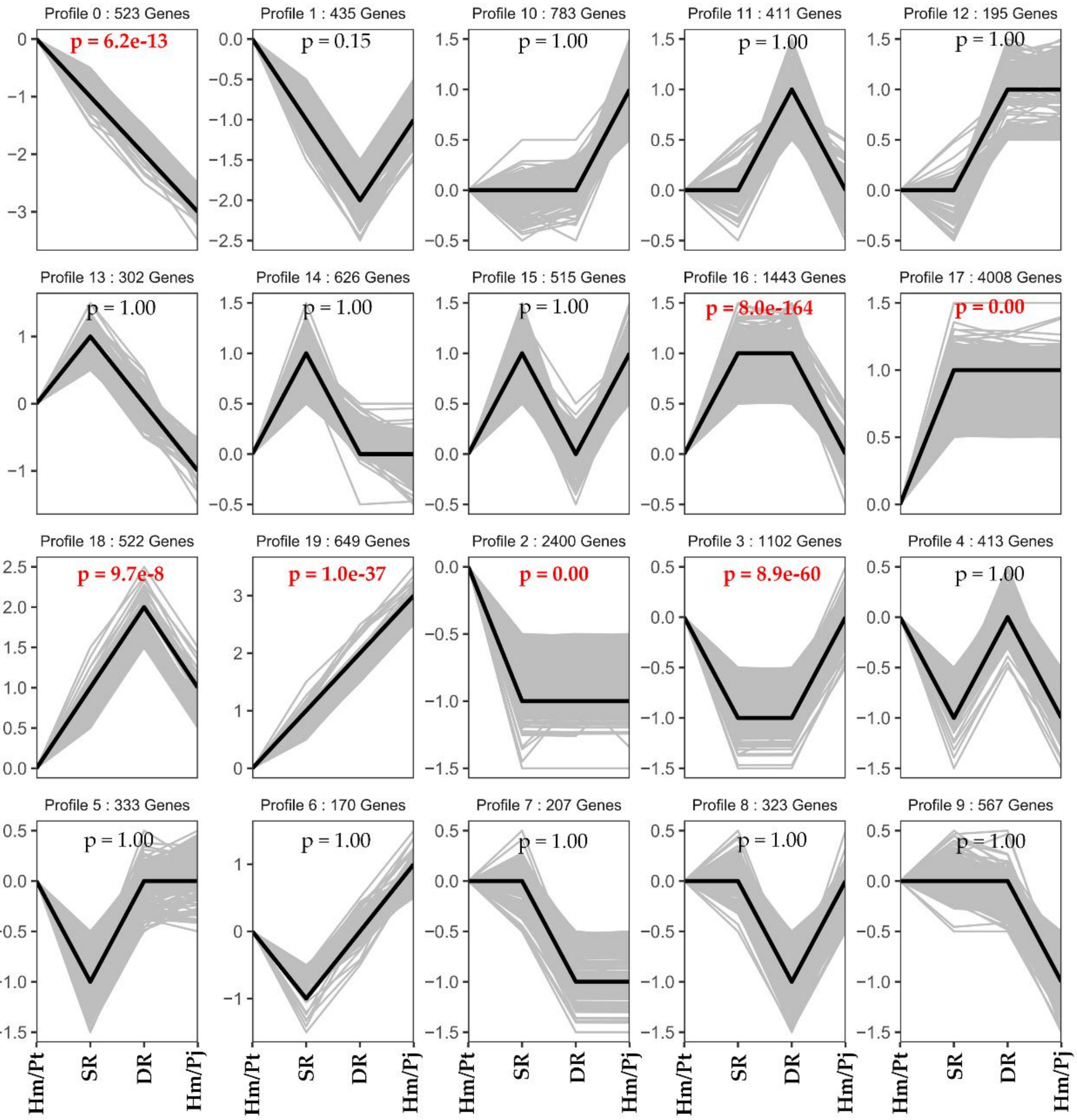

Figure S1. Graft-induced transcripts grouped according to temporal expression profiles. Each of the profiles is represented as a different plot.

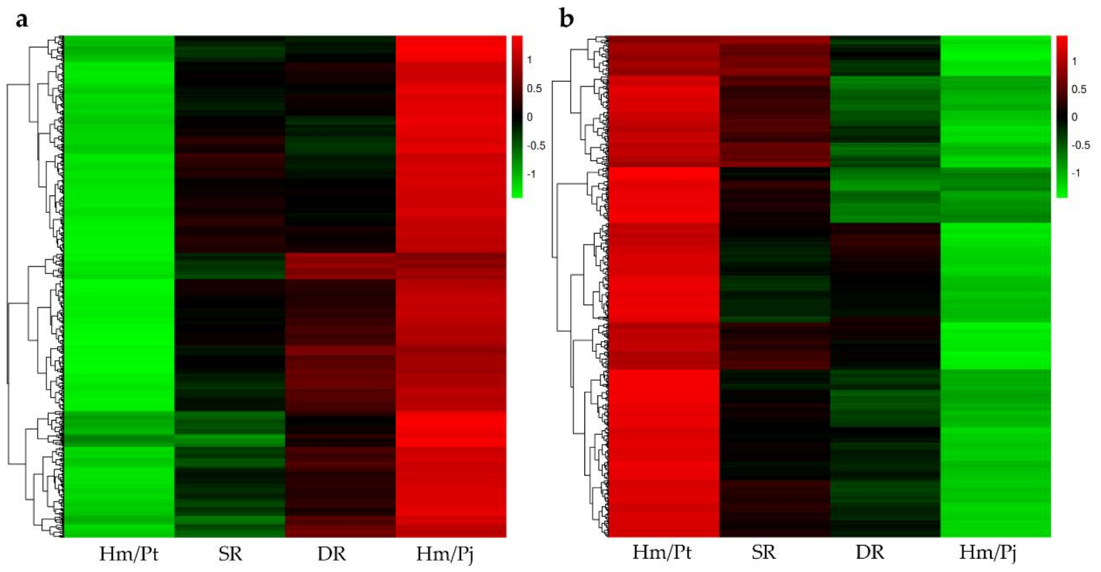

Figure S2. Heatmap of genes in upregulated and downregulated expression profiles. Heatmap color indicates $\log_{10}^{(FPKM+0.01)}$ value. **a** Heatmap of genes in profile 19, **b** Heatmap of genes in profile 0.

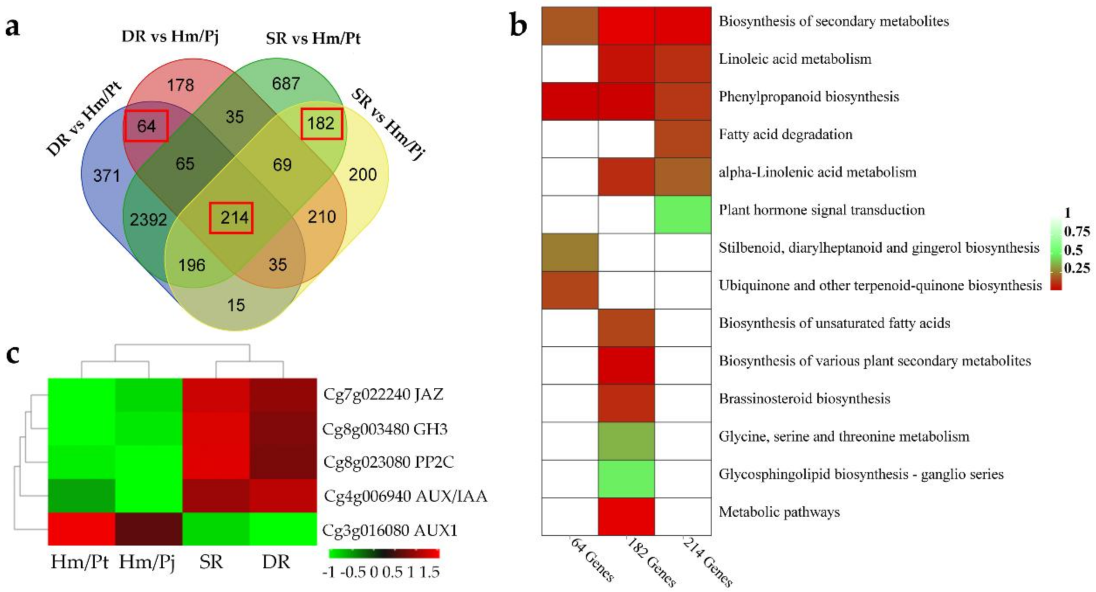

Figure S3. DEG analysis between different graft combinations. a Venn diagram of DEGs in each graft combinations. **b** KEGG pathways for different compare groups. c heatmap of DEGs between DR and SR vs Hm/Pt and Hm/Pj.

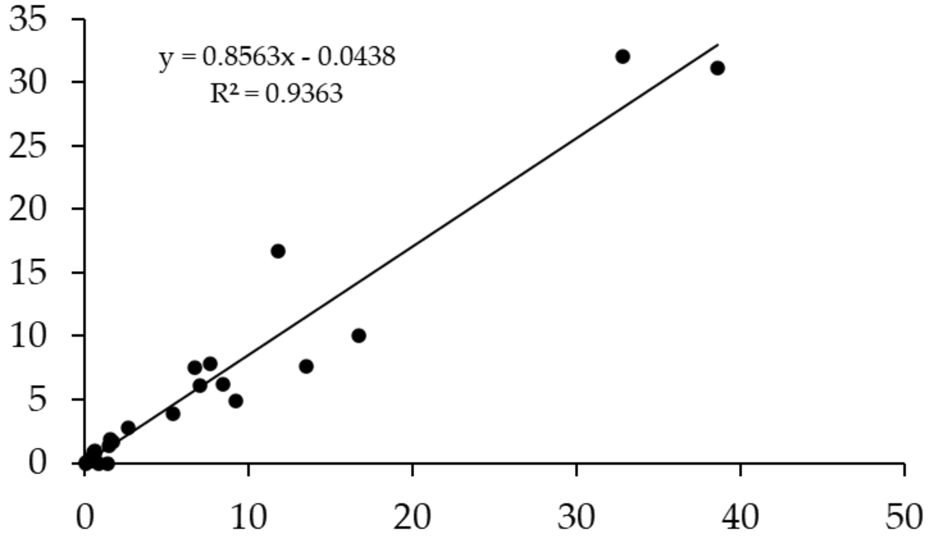

Figure S4. Pearson's correlation of RNA-seq and qRT-PCR results.

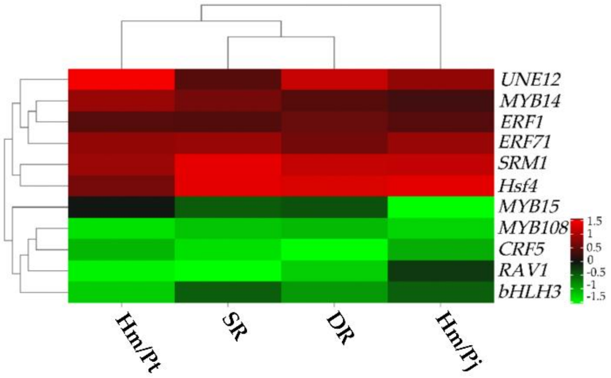

Figure S5. The expression of important graft compatibility related transcription factors. Heatmap color indicates $\log_{10}$(FPKM+0.01) value.
